# Clinical Audit of Use of Clonazepam in General Adult Psychiatry Patients

**DOI:** 10.1192/bjo.2025.10581

**Published:** 2025-06-20

**Authors:** Nizar El Ahmadie, Constance Sturgess, Jamshid Nazari

**Affiliations:** South West Yorkshire NHS Foundation Trust, Wakefield, United Kingdom

## Abstract

**Aims:** To assess adherence to local prescribing guidelines for clonazepam, including indication appropriateness, consultant oversight, documentation of unlicensed medicine forms, weekly review compliance, and discontinuation planning before discharge.

**Methods:** A retrospective audit was conducted on the electronic records of 234 patients under the care of the Enhanced Team West (ETW) between July 2023 and August 2024. Using a keyword search for “clonazepam”, 20 patients were identified, with 16 meeting inclusion criteria. Data on demographics, indications, prescribing teams, consultant involvement, documentation of unlicensed medicine forms, review frequency, and discontinuation plans were collected and analysed.

**Results:** Indication compliance: 94% of prescriptions were for appropriate indications, predominantly agitation (56%) and anxiety (19%). No cases were for epilepsy.

Consultant oversight: 88% of prescriptions were initiated or approved by a consultant psychiatrist.

Unlicensed medicine form usage: Only 6% of cases had a completed unlicensed medicine form, despite trust guidelines mandating its use.

Review frequency: Weekly reviews were documented in 100% of cases, primarily through multidisciplinary team (MDT) meetings.

Discontinuation planning: 47% of patients had a documented plan to taper or discontinue clonazepam before discharge. Among those continued post-discharge, 89% had documented attempts at dose reduction in the community.

**Conclusion:** This audit highlights high compliance with appropriate indications and consultant oversight but identifies areas for improvement in documentation of unlicensed medicine forms and structured discontinuation planning. Given the risks associated with prolonged clonazepam use, enhancing adherence to trust guidelines on discontinuation strategies is essential. Recommendations include improved training on unlicensed medicine documentation, reinforced discharge planning protocols, and regular re-audits to monitor adherence and patient outcomes.

